# ClusterCAD: a computational platform for type I modular polyketide synthase design

**DOI:** 10.1093/nar/gkx893

**Published:** 2017-10-11

**Authors:** Clara H Eng, Tyler W H Backman, Constance B Bailey, Christophe Magnan, Héctor García Martín, Leonard Katz, Pierre Baldi, Jay D Keasling

**Affiliations:** Department of Chemical and Biomolecular Engineering, University of California, Berkeley, CA 94720, USA; Joint BioEnergy Institute, 5885 Hollis Street, Emeryville, CA 94608, USA; Biological Systems and Engineering Division, Lawrence Berkeley National Laboratory, Berkeley, CA 94720, USA; Department of Energy Agile BioFoundry, Emeryville, CA 94608, USA; Department of Computer Science, University of California, Irvine, CA 92697, USA; Institute for Genomics and Bioinformatics, University of California, Irvine, CA 92697, USA; QB3 Institute, University of California, Berkeley, CA 94720, USA; Department of Bioengineering, University of California, Berkeley, CA 94720, USA; Novo Nordisk Foundation Center for Biosustainability, Technical University of Denmark, DK2970 Horsholm, Denmark

## Abstract

ClusterCAD is a web-based toolkit designed to leverage the collinear structure and deterministic logic of type I modular polyketide synthases (PKSs) for synthetic biology applications. The unique organization of these megasynthases, combined with the diversity of their catalytic domain building blocks, has fueled an interest in harnessing the biosynthetic potential of PKSs for the microbial production of both novel natural product analogs and industrially relevant small molecules. However, a limited theoretical understanding of the determinants of PKS fold and function poses a substantial barrier to the design of active variants, and identifying strategies to reliably construct functional PKS chimeras remains an active area of research. In this work, we formalize a paradigm for the design of PKS chimeras and introduce ClusterCAD as a computational platform to streamline and simplify the process of designing experiments to test strategies for engineering PKS variants. ClusterCAD provides chemical structures with stereochemistry for the intermediates generated by each PKS module, as well as sequence- and structure-based search tools that allow users to identify modules based either on amino acid sequence or on the chemical structure of the cognate polyketide intermediate. ClusterCAD can be accessed at https://clustercad.jbei.org and at http://clustercad.igb.uci.edu.

## INTRODUCTION

Polyketides and their derivatives are used broadly in medicine as antibiotics, antifungals and immunosuppressants, among other applications (1), motivating research to engineer polyketide synthases (PKSs) capable of producing novel drug analogs. More recently, researchers have also harnessed the biosynthetic potential of PKSs to produce commodity chemicals in an environmentally friendly manner (2–4).

**Figure 1.**
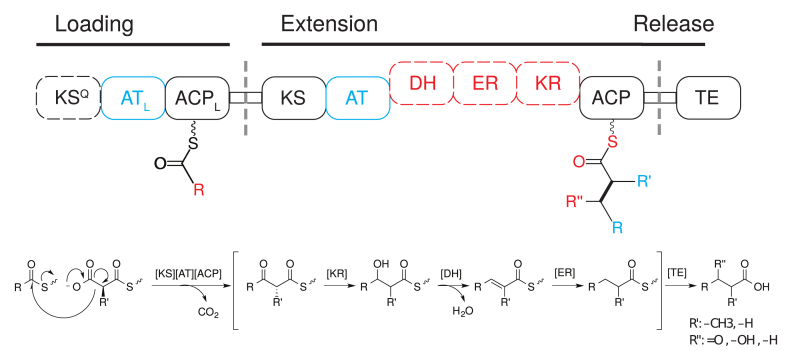
Determination of polyketide chemical structure by a sequence of PKS catalytic domains. A loading AT_L_ (blue) selects a starter acyl-CoA and catalyzes the chain initiation reaction, thereby specifying the identity of the R group in the extension reaction product. This acyl-intermediate is then transferred to the KS. The AT (blue) selects a malonyl-CoA derivative as an extender unit, which is subsequently transferred to the ACP, determining the R′ group in the extension reaction product. Next, the KS catalyzes a decarboxylative condensation between the KS bound acyl-intermediate and the ACP-bound extender unit to yield an ACP-tethered product, with the newly formed C-C bond shown in bold. The ketone located at the β position of the resulting intermediate may be optionally reduced by a KR to form a hydroxyl group, a DH to form an methenyl group and an ER to form methylene group. The presence or absence of these reductive domains (red) therefore determine the identity of the R″ group. This poly-β-ketone may go through multiple extension steps before reaching a TE, which catalyzes either hydrolysis or cyclization to form an acid or a lactone, respectively.

Type I modular PKSs are composed of a series of catalytic domains whose identity and order determine the structure of the final polyketide product in a predictable manner, as shown in Figure 1. A single PKS can include multiple polypeptide subunits, each comprising one or more modules. Each module, which is minimally made up of a set of ketosynthase (KS), acyltransferase (AT) and acyl carrier protein (ACP) domains, catalyzes the extension of the nascent acyl chain by two carbon units. This is accomplished via a KS-catalyzed decarboxylative condensation between an acyl starter or intermediate, and a dicarboxylic acid extender unit, which is selected by the AT (5,6). The resulting β-ketone may be reduced by a ketoreductase (KR) domain, which determines the stereochemistry of the resulting β-hydroxyl group, and that of the α-substituent, if present (7–11). Additional dehydratase (DH) and enoyl reductase (ER) domains may further reduce the hydroxyl group to yield a methenyl or methylene group, respectively. The KR, DH and ER make up a set of optional reducing domains that together, are termed the reductive cassette. In addition to these reductive domains, C- or O- methyltransferases may also be present. Release of a polyketide from a PKS is carried out by a thioesterase (TE) domain that catalyzes either hydrolysis or cyclization. The resulting acid or lactone may undergo additional modifications to form the final active compound (12).

The potential interchangeability of PKS catalytic domains make PKSs an appealing prospective toolkit for combinatorial biosynthesis. The structure of the two-carbon addition to the nascent acyl chain that is effected by a single PKS module is determined by the substrate specificity of the AT domain, the stereochemical outcome modulated by the KR domain, and the number of active reducing domains in the reductive cassette. Over 20 different malonyl-CoA analogs have been observed to be used as extender units in naturally occurring PKSs (3,13,14). Furthermore, all possible combinations of stereochemical configurations at the α and β positions are made accessible by different KR domains. Developing methods to effectively recombine existing PKSs thus offers a tantalizing promise of access to an expansive region of chemical space.

However, in spite of recent structural and mechanistic insights gained from structural biology techniques, including X-ray crystallography (15–17) and cryo-electron microscopy (18,19), a comprehensive model of the protein–protein interactions and catalytic mechanisms that govern the fold and function of PKS modules remains elusive. Nevertheless, encouraging earlier work has successfully combined intuition and expert domain knowledge with inspection of existing sequence alignments and X-ray crystal structures to engineer chimeric PKSs harboring a heterologous AT domain (3,20–22), a heterologous KR domain (23–25), or a heterologous reductive cassette (2,21,24,26–28). These results suggest that it is possible to identify combinations of junctions and catalytic domains that facilitate the construction of active PKS chimeras. While codifying the intuition that has previously enabled successful domain exchanges is impossible, one commonly used heuristic is to seek to design a chimeric PKS that is as similar to a naturally occurring PKS as possible. Using this guiding principle, we propose the following paradigm for designing a chimeric PKS capable of producing a small molecule compound of interest:

- Identify a truncated PKS parent to use as a starting point for engineering by searching for a module known to produce a polyketide intermediate with maximal structural similarity to the compound of interest.
- Decompose the difference between the natural product of the truncated PKS starting point and the compound of interest into a series of catalytic domain exchanges.
- Select donor modules for the required catalytic domain exchanges on the basis of sequence similarity to the truncated PKS parent.

ClusterCAD was developed with the aim of enabling the design of chimeric PKSs following this paradigm. As such, it includes a structural search tool to facilitate the identification of a truncated PKS parent to use as an engineering starting point given a small molecule target, and a sequence search tool to aid in the selection of donor modules for catalytic domain exchanges based on sequence similarity to the truncated PKS parent. However, we emphasize that ClusterCAD is intended to augment, rather than supersede, the biochemical intuition of PKS synthetic biologists.

Sequence similarity between PKS modules, and structural similarity between their cognate polyketide intermediates, provide only a rudimentary method of predicting compatibility between PKS polypeptide sequences. It is thus also important to consider additional factors when designing a chimeric PKS. For example, modules that originate from well-characterized clusters, or that have been previously determined to be well-expressed in the host organism of choice, are particularly attractive choices for engineering.

## MATERIALS AND METHODS

### Data curation

The gene clusters in ClusterCAD are sourced from the Minimum Information about a Biosynthetic Gene cluster (MIBiG) database (29). To construct each ClusterCAD entry, the gene clusters in version 1.3 (3 September 2016) of the MIBiG database labeled as type I modular PKSs were first identified. These gene clusters were annotated using the antibiotics and Secondary Metabolite Analysis SHell (antiSMASH) (30), and the resulting output parsed using a list of explicitly recognized catalytic domains. Cluster analysis was truncated if a subunit containing an unrecognized catalytic domain was encountered. Domain annotations, which include predictions for AT substrate specificity and KR stereochemical outcome, informed *in silico* simulation of biocatalysis using reaction SMARTS operators (http://www.daylight.com/dayhtml/doc/theory/), which were implemented using the RDKit cheminformatics software library (http://www.rdkit.org), yielding a predicted intermediate chemical structure for each PKS module. Known final chemical structures were taken from the MIBiG database if available, or were otherwise generated from the text description of the final structure in the MIBiG database using the ChemAxon JChem Base naming tool (version 17.2.27.0, 2017) (31). Chemical structures for well-characterized polyketide products that could not be obtained by either of these methods were manually incorporated. Comparisons of the predicted and known final product chemical structures, close inspection of established amino acid sequence motifs (20,32) and an extensive literature search were used to manually curate each ClusterCAD entry by correcting subunit order, removing invalid subunits, accounting for iterating modules and correcting misannotated catalytic domain activity. The SCRATCH software suite (33,34) was used to generate secondary structure and relative solvent accessibility predictions for the PKS subunits in ClusterCAD, which were also incorporated into the database.

### Organization

#### User interface

**Figure 2.**
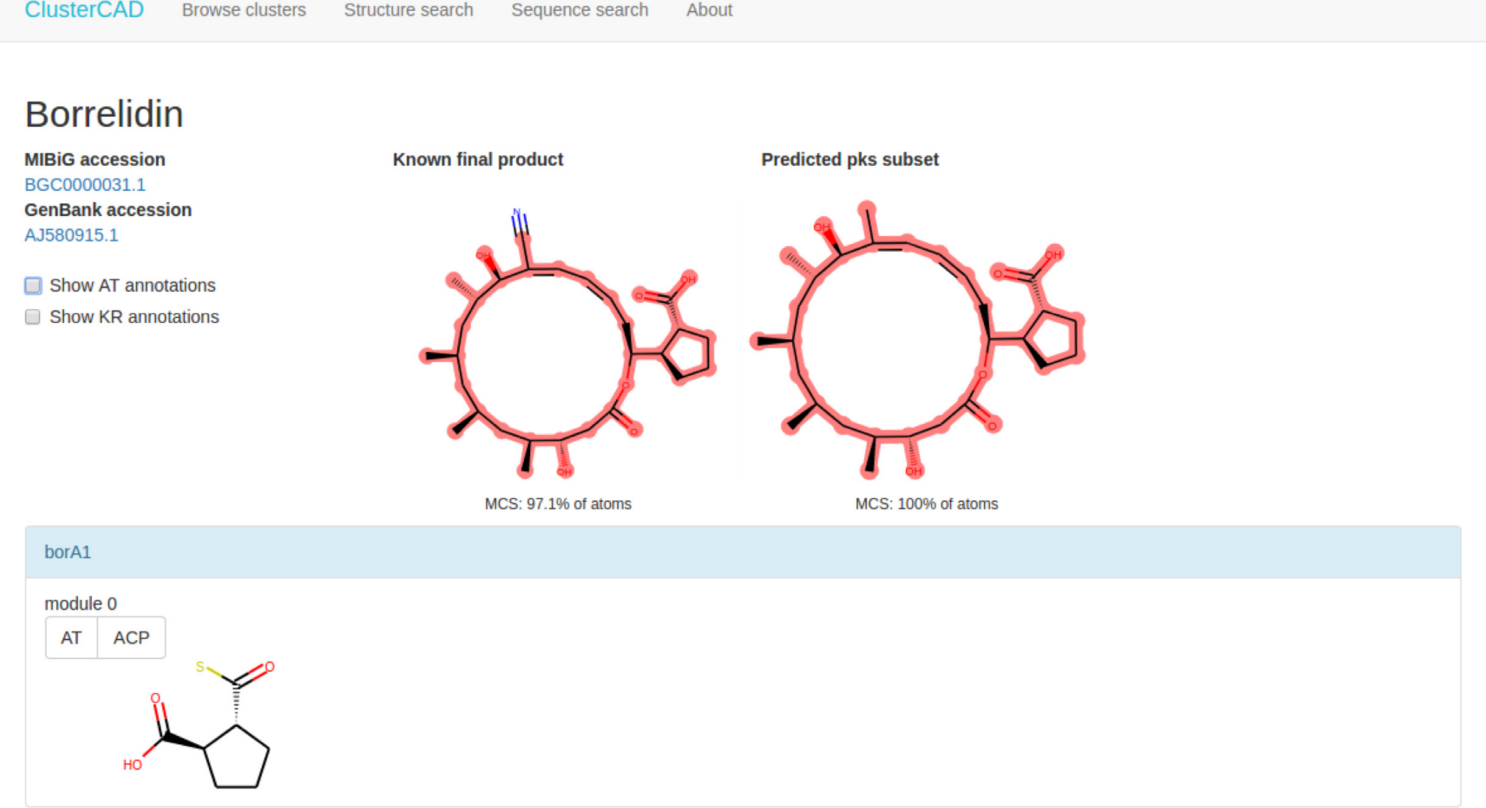
ClusterCAD entry for the borrelidin PKS. The ClusterCAD page for each cluster contains links to the corresponding entries in the MiBiG and NCBI Nucleotide databases. Catalytic domain annotations can be optionally displayed based on domain type. Interactive web page elements provide access to more detailed information, such as the nucleotide and amino acid sequences of each catalytic domain and subunit.

The primary objective of ClusterCAD is to provide computational assistance to the synthetic biologist seeking to engineer PKSs. ClusterCAD provides an intuitive interface for users to browse through the clusters in the database, as well as search tools developed to assist in the design of chimeric PKSs. An example entry of a PKS cluster as displayed in the ClusterCAD web interface, is shown in Figure 2.

- *Structure search tool*: the structure search tool was designed to enable the identification of a truncated PKS parent to use as a starting point for PKS engineering, and takes as input either a small molecule chemical structure represented as a SMILES string, or a structure that is drawn in an interactive graphical user interface. This tool searches a database of the predicted polyketide intermediates produced by each of the PKS modules in ClusterCAD. Matches to the query structure are ranked using atom pair (AP) Tanimoto similarity scores (35), and returned matches are displayed with the maximum common substructure (36) between the query and target structures highlighted.
- *Sequence search tool*: the sequence search tool was designed to enable researchers to select candidate catalytic domains for domain exchange experiments, and supports a variety of queries that allow researchers to test hypotheses regarding which domain–domain interactions may be important in facilitating successful domain exchanges. The sequence search tool takes as input an amino acid sequence, and performs a protein–protein BLAST+ search against a database containing all of the subunits in ClusterCAD (37). The results page enables deeper interrogation of the returned results by highlighting catalytic domains on the basis of annotations such as AT substrate specificity, KR stereochemical outcome, and reductive domain activity.

## DISCUSSION

### Motivation behind ClusterCAD

ClusterCAD provides the first PKS database that specifically targets engineering applications. Existing PKS databases focus on identifying new metabolites through genome mining or on predicting metabolite structure from nucleotide or amino acid sequence (38). However, many of the most promising applications of PKSs are premised on harnessing their ability to generate customized molecular architectures.

From an engineering perspective, ClusterCAD offers substantial improvements over existing databases. By taking advantage of resources such as the MIBiG database and the antiSMASH command-line tool for cluster identification and annotation respectively, we were able to focus on designing tools tailored to the needs of the synthetic biologist without attempting to optimize our software for genome mining or metabolite structure prediction. Furthermore, given the strong commitment from the synthetic biology and natural products communities to update MiBiG and antiSMASH with continuous improvements, populating ClusterCAD using an end-to-end pipeline that utilizes these tools means that ClusterCAD can easily be kept up-to-date as novel biosynthetic clusters are discovered and as our understanding of PKSs develops.

While building on MiBiG and antiSMASH aids in automating the maintenance of ClusterCAD, it also means that inaccuracies in MiBiG or in the antiSMASH software may be propagated into ClusterCAD. In order to address this issue, we also employ extensive manual curation of each ClusterCAD entry in order to provide predicted polyketide intermediates that are as accurate as possible. ClusterCAD has a persistent corrections record that overwrites the output generated by antiSMASH. This enables ClusterCAD to incorporate new MiBiG entries and systematic improvements to antiSMASH, while also guaranteeing that clusters that have previously been manually corrected will remain correct. Because these records are persistent and override the automatically generated output, the manually curated clusters will only need to be further corrected if new empirical findings are generated. ClusterCAD thus combines computational annotations of AT substrate specificity and KR stereospecificity with domain expert application of heuristics obtained from the literature (20,32,39,40) to provide well-curated predictions of the polyketide intermediates naturally provided by each of the PKS modules in ClusterCAD. However, the reliance on manual curation means that some errors may be missed. We include links on each cluster page that can be used to submit corrections to the ClusterCAD maintainers for review.

ClusterCAD is further limited in that it does not support inclusion of chemistry effected by ‘on-line’ tailoring enzymes that act on ACP-tethered polyketide intermediates, but that are independent of the PKS (41). However, we believe that showing the direct product of the PKS, without taking into account ‘on-line’ tailoring enzymes provides the information that is most relevant for engineering, since ‘on-line’ tailoring enzymes are not expected to be naturally present in a heterologous host.

A substantial benefit of ClusterCAD is that it provides engineering tools that allow researchers to compare PKS modules based on amino acid sequence similarity and on cognate polyketide intermediate similarity. The substructure search in ClusterCAD is of particular utility when selecting candidate modules to serve as a starting point in a PKS engineering project. Previous experimental evidence supports the assertion that the structure of the intermediate can have a significant impact on the function of individual catalytic domains. KS domains, for example, have been reported to have ‘gatekeeping’ functionality, in which they are unable to condense intermediates that are chemically dissimilar for reasons ranging from overall steric bulkiness (42) to having an opposite stereochemical configuration (43,44). Other domains, such as the KR domain (25) and the DH domain (45) have also been observed to have poor catalytic activity on substrates that are much smaller or bulkier than ones they encounter in nature.

The substructure search in ClusterCAD is one of the first that matches against polyketide intermediates associated with non-terminal PKS modules, making it more robust to false positives than similar search tools which only support substructure queries in the context of PKS final products (46). Matching directly against the polyketide intermediates produced by each module also avoids undesirable matches to structural motifs produced by tailoring enzymes, which are of limited utility for PKS engineering. To the best of our knowledge, SBSPKSv2 (47) is the only other PKS database that supports structure-based queries that match against polyketide intermediates. However, ClusterCAD boasts a larger number of type I modular PKS intermediates, and utilizes AP descriptors, which exhibit greater sensitivity to uncommon chemical motifs than the substructure fingerprint approach used by SBSPKSv2.

Further, anecdotal evidence supports the assertion that seeking catalytic domains with greater sequence similarity improves domain exchange success rates (25). While this heuristic is challenging to verify experimentally given the low throughput of domain exchange experiments, it is our hope that ClusterCAD will assist PKS researchers in elucidating the importance of sequence similarity in domain exchange experiments by streamlining the design of experiments to test their hypotheses. For instance, by facilitating chimeric PKS design, ClusterCAD will enable researchers to probe potential domain–domain interactions which may be important for engineering.

The sequence search tool in ClusterCAD highlights the domains in each returned module that overlap with the query, and also allows the user to highlight results based on catalytic domain activity. Identifying junctions in PKS chimeras that reliably leave functionally important structural elements intact (24,25) remains an open area of research. ClusterCAD provides secondary structure and relative solvent accessibility predictions that will aid in the identification and comparison of homologous structural elements across PKSs, assisting researchers in junction design. As far as we are aware, ClusterCAD is the only PKS database that provides precomputed values for the predicted secondary structure and relative solvent accessibility of each subunit. By providing support to compare PKS modules on the basis of metrics that may contribute to compatibility between PKS polypeptide sequences, ClusterCAD aims to facilitate experimental research to identify robust strategies for PKS engineering.

### Using ClusterCAD to design a chimeric PKS that produces adipic acid

While engineered PKSs have long been an attractive strategy to produce pharmaceutically relevant natural products and their analogs, more recent work has also sought to harness the chemical diversity accessible by PKS to provide an environmentally friendly alternative to chemical synthesis for commodity chemicals. Previous work by Hagen *et al.* has demonstrated that it is possible to engineer a novel PKS capable of producing adipic acid (2), a widely used commodity chemical. As a proof-of-concept, we describe how ClusterCAD could be applied to engineer this novel PKS.

- *Identify a truncated PKS starting point that naturally produces a polyketide intermediate that is structurally similar to adipic acid*. Hagen *et al.* selected the first module from the borrelidin PKS as a starting point based on previous biochemical studies establishing that BorMod1 tolerates succinyl-CoA as a starter, possibly because of structural similarity to the native priming unit, trans-1,2-cyclopentanedicarboxylic acid (48). Adipic acid was used to query the ClusterCAD chemical structure search tool, which returned BorMod1 as the highest-scoring result, with a naturally produced intermediate that had an AP Tanimoto similarity score of 0.32 when compared against adipic acid.
- *Identify a donor reductive loop with active ER, DH and KR from a module which naturally incorporates a malonyl-CoA extender unit*. In order to produce adipic acid using BorMod1, incorporation of an extender unit lacking a α-substituent and reduction by a full complement of active reducing domains was required. Hagen *et al.* thus chose four reductive loop donors that naturally accepted malonyl-CoA, harbored a DH, ER and KR in the reductive cassette, and that came from a ‘standalone’ module, in which the corresponding open reading frame or subunit contained only a single module. Donor loops from ‘standalone’ modules were favored based on the intuition that domain exchanges using reductive cassettes derived from a ‘standalone’ module would retain more architectural similarity to BorMod1, itself a ‘standalone’ module, and thereby increase the likelihood of a functional chimera.
  Using the ClusterCAD sequence search tool, we sought to identify donor modules that not only met these criteria, but also exhibited maximal sequence similarity to BorMod1. We queried the ClusterCAD sequence search tool with the amino acid sequence of BorA2, the subunit which contains BorMod1, ranking the potential donor modules by bit score and filtering for ‘standalone’ modules containing a malonyl-CoA specific AT and a complete reductive cassette. IdmO, from the indanomycin PKS, followed by SpnB from the spinosyn PKS were the only two returned results that matched all three criteria. Chimeras utilizing these two donor modules were experimentally tested by Hagen *et al.*, along with chimeras harboring reductive cassettes from the donor modules AurB, from the aureothin PKS and NanA2, from the nanchangmycin PKS. AurB, which is not contained in ClusterCAD, and SpnB were empirically determined to yield the most active chimeras. NanA2, which was not returned by ClusterCAD, but is contained in the database, yielded the least active chimera.
- *Identify a DH domain that exhibits higher levels of activity on 3-hydroxy adipic acid*. The chimeras constructed by Hagen *et al.* yielded a relatively large proportion of the incompletely reduced product 3-hydroxyadipic acid, potentially due to the DH from the donor reductive cassettes having a poor substrate compatibility with the terminal carboxyl moiety, an unusual feature in PKS biosynthetic intermediates. To overcome this substrate incompatibility, Hagen *et al.* further substituted a heterologous DH into the engineered BorMod1 variant. The DH from BorMod2 was selected based on the observation that it is known to demonstrate catalytic activity on a substrate containing a terminal carboxy moiety. The intuition employed by Hagen *et al.* was consistent with the sequence-similarity based output of the ClusterCAD search tool, which returned BorMod2 as the module containing an active DH that had the highest bit score with BorMod1.

### Implications of ClusterCAD for PKS engineering

**Figure 3.**
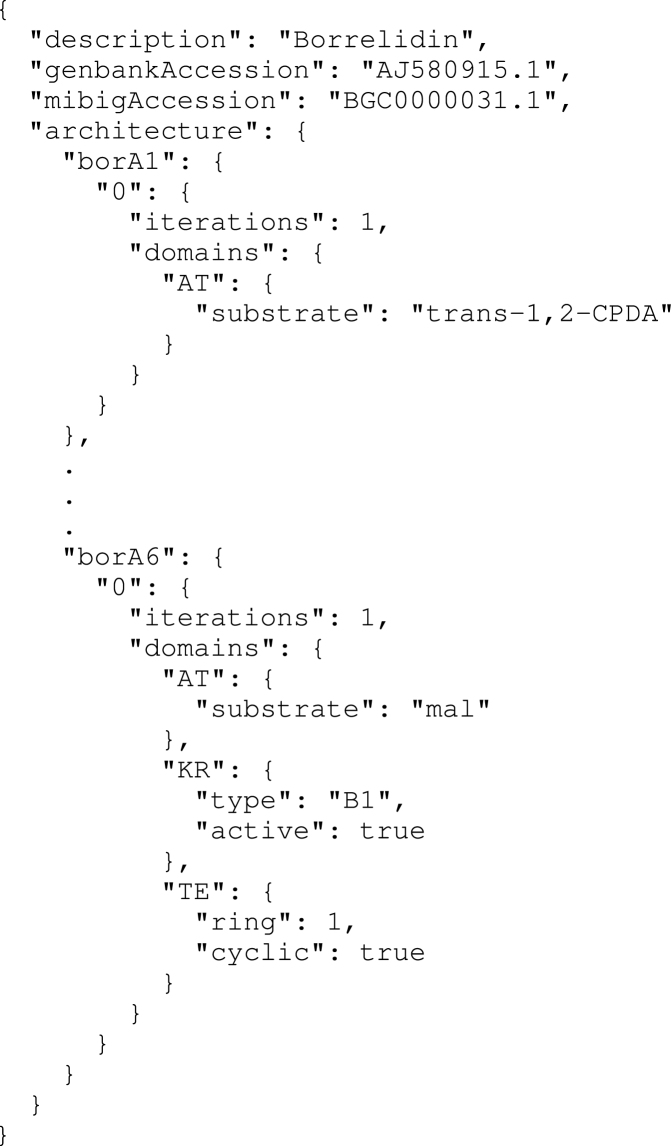
Partial JSON representation of the borrelidin PKS. This representation captures the hierarchical organization of PKSs, as well as the important catalytic properties of their components.

As the first PKS database that specifically targets synthetic biologists interested in PKS engineering, we also sought more generally to provide tools for computer-aided PKS design, and have released ClusterCAD under a BSD style license at https://github.com/JBEI/clusterCAD. This open source release provides Python object representations of catalytic domains, modules and clusters, that enable researchers to construct and simulate the activity of chimeric PKSs *in silico*. Furthermore, we sought to establish a method to describe PKSs in a human and computer readable manner using the JSON data-interchange format, as shown in Figure 3. The availability of such a PKS representation will aid in the development of additional software for computational PKS design by allowing researchers to easily describe novel PKSs in a computer-readable fashion.

## CONCLUSION

To the best of our knowledge, ClusterCAD is the first web-based toolkit developed specifically to aid in designing chimeric PKSs. By providing chemical structures with stereochemistry for the polyketide intermediate produced by each module, as well as relative solvent accessibility and secondary structure predictions for each subunit, we provide rich information about each cluster to inform chimeric PKS design. Furthermore, our intuitive user interface, combined with the ability to query for modules based on either sequence- or structure-similarity provide powerful tools for PKS engineering that bring us closer to the promise of harnessing PKSs for combinatorial biosynthesis.
